# Glucose-6-phosphate dehydrogenase deficiency and the risk of malaria and other diseases in children in Kenya: a case-control and a cohort study

**DOI:** 10.1016/S2352-3026(15)00152-0

**Published:** 2015-09-22

**Authors:** Sophie Uyoga, Carolyne M Ndila, Alex W Macharia, Gideon Nyutu, Shivang Shah, Norbert Peshu, Geraldine M Clarke, Dominic P Kwiatkowski, Kirk A Rockett, Thomas N Williams

**Affiliations:** a Department of Epidemiology and Demography, KEMRI–Wellcome Trust Research Programme, Kilifi, Kenya; b Wellcome Trust Centre for Human Genetics, University of Oxford, Oxford, UK; c Laboratory of Malaria and Vector Research, National Institute of Allergy and Infectious Diseases, National Institutes of Health, Bethesda, MD, USA; d Wellcome Trust Sanger Institute, Hinxton, Cambridge, UK; e Department of Medicine, Imperial College, St Mary's Hospital, London, UK

## Abstract

**Background:**

The global prevalence of X-linked glucose-6-phosphate dehydrogenase (G6PD) deficiency is thought to be a result of selection by malaria, but epidemiological studies have yielded confusing results. We investigated the relationships between G6PD deficiency and both malaria and non-malarial illnesses among children in Kenya.

**Methods:**

We did this study in Kilifi County, Kenya, where the G6PD c.202T allele is the only significant cause of G6PD deficiency. We tested the associations between G6PD deficiency and severe and complicated *Plasmodium falciparum* malaria through a case-control study of 2220 case and 3940 control children. Cases were children aged younger than 14 years, who visited the high dependency ward of Kilifi County Hospital with severe malaria between March 1, 1998, and Feb 28, 2010. Controls were children aged between 3–12 months who were born within the same study area between August 2006, and September 2010. We assessed the association between G6PD deficiency and both uncomplicated malaria and other common diseases of childhood in a cohort study of 752 children aged younger than 10 years. Participants of this study were recruited from a representative sample of households within the Ngerenya and Chonyi areas of Kilifi County between Aug 1, 1998, and July 31, 2001. The primary outcome measure for the case-control study was the odds ratio for hospital admission with severe malaria (computed by logistic regression) while for the cohort study it was the incidence rate ratio for uncomplicated malaria and non-malaria illnesses (computed by Poisson regression), by G6PD deficiency category.

**Findings:**

2863 (73%) children in the control group versus 1643 (74%) in the case group had the G6PD normal genotype, 639 (16%) versus 306 (14%) were girls heterozygous for G6PD c.202T, and 438 (11%) versus 271 (12%) children were either homozygous girls or hemizygous boys. Compared with boys and girls without G6PD deficiency, we found significant protection from severe malaria (odds ratio [OR] 0·82, 95% CI 0·70–0·97; p=0·020) among G6PD c.202T heterozygous girls but no evidence for protection among G6PD c.202T hemizygous boys and homozygous girls (OR 1·18, 0·99–1·40; p=0·056). Median follow-up for the mild disease cohort study was 2·24 years (IQR 2·22–2·85). G6PD c.202T had no effect on other common diseases of childhood in heterozygous girls (incidence rate ratio 0·98, 95% CI 0·86–1·11; p=0·82) or homozygous girls or hemizygous boys (0·93, 0·82–1·04; p=0·25), with the sole exception of a marginally significant increase in the incidence of helminth infections among heterozygous girls.

**Interpretation:**

Heterozygous girls might be the driving force for the positive selection of G6PD deficiency alleles. Further studies are needed to definitively establish the mechanisms by which G6PD deficiency confers an advantage against malaria in heterozygous individuals. Such studies could lead to the development of new treatments.

**Funding:**

Wellcome Trust, UK Medical Research Council, European Union, and Foundation for the National Institutes of Health (as part of the Bill & Melinda Gates Grand Challenges in Global Health Initiative).

## Introduction

Glucose-6-phosphate dehydrogenase (G6PD) plays an important role in the body's defence against oxidant damage that is particularly important in red blood cells.^1^ The X-chromosome-linked G6PD gene is one of the most polymorphic loci of human beings. Almost 200 allelic variants have been reported, of which around 40 have reached polymorphic frequencies in multiple populations,^2^ which is widely thought to be a result of natural selection by malaria.^3^ Nevertheless, epidemiological studies investigating this hypothesis have yielded confusing results that have not always supported this conclusion. A particular controversy has surrounded the question of whether protection is extended to both boys and girls or is exclusive to female carriers.^4^ We investigated the effect of G6PD deficiency on a range of malaria-specific and non-malaria outcomes in children living in an area in which the G6PD c.202T allele is the only significant genetic cause.^5^

**Research in context**

**Evidence before this study**

We systematically searched PubMed up to May 22, 2015, for original research articles investigating the relationship between G6PD deficiency and malaria, using the term “((glucose-6-phosphate dehydrogenase deficiency) AND malaria) OR (G6PD AND malaria) NOT “review”[publication type]”. Our search returned 564 articles, which we supplemented with secondary citations and our personal collections. Most reports focused on drug treatment of malaria, particularly the effects of primaquine and other antimalarial drugs in people with G6PD deficiency, on diagnostic methods for detecting G6PD deficiency, on G6PD deficiency as a cause of blackwater fever, or were limited to descriptions of the prevalence of G6PD deficiency, and included data from studies from diverse regions with very different genetic backgrounds and malaria epidemiology. We therefore restricted our search to studies done in Africa by the inclusion of the additional search term “AND Africa”, returning 157 articles that were subjected to further appraisal. These studies used a wide range of experimental designs and most had a small sample size (<200); however, 34 reports contained at least some data regarding the association between G6PD deficiency and malaria from clinical or epidemiological studies since 1960, of which eight contained data on asymptomatic parasitaemia, 15 contained data on uncomplicated malaria, and 16 contained data on severe or complicated malaria. These reports reached a range of conclusions about the relationship between G6PD deficiency and malaria, including no significant effect (six reports) or positive or negative associations in various groups of males or females (28 reports). Some studies have considered heterogeneity at the G6PD locus as a potential explanation for the confusing findings from earlier studies, concluding that G6PD deficiency is a balanced polymorphism driven by selection for heterozygous females. The wide range of study designs, diagnostic approaches, and populations under study precluded a formal meta-analysis.

**Added value of this study**

We investigated the effects of G6PD deficiency on clinical malaria phenotypes ranging from asymptomatic parasitaemia to strictly defined severe malaria, in 7031 children in a malaria-endemic region of Kenya. We considered allelic heterogeneity at the G6PD locus and the possibility of interactions with other common malaria-protective red cell polymorphisms. We found that the effects of G6PD deficiency were limited to severe malaria and malaria-specific mortality, adding new weight to the conclusion that G6PD deficiency is a balanced polymorphism favouring heterozygous girls.

**Implications of the available evidence**

Further studies are needed to definitively establish the mechanisms by which G6PD deficiency confers an advantage against malaria in heterozygous individuals. Such studies could lead to the development of new treatments. G6PD deficiency has different effects against different subtypes of severe malaria, predisposing to severe malarial anaemia but protecting against cerebral malaria. Moreover, these subtypes vary with malaria transmission intensity and thus between populations and over time, meaning that the net evolutionary pressures for G6PD deficiency might be more complex than previously appreciated.

## Methods

### Study design and participants

We examined the effect of G6PD deficiency on the risk of malaria and other childhood diseases in children living within the area served by the Kilifi Health and Demographic Surveillance System in Kenya.^6^ We did the study in two parts: first, we did a case-control study to assess the effect of G6PD deficiency on the risk of severe *Plasmodium falciparum* malaria; second, we did a cohort study to investigate its effect on the risk of uncomplicated clinical malaria and other common diseases of childhood.

For the case-control part of the study, we included children aged younger than 14 years who were admitted with clinical features of severe or complicated *P falciparum* malaria to the high dependency ward of Kilifi County Hospital between March 1, 1998, and Feb 28, 2010, as cases. Severe malaria was defined by blood-film positivity for *P falciparum* and the patient having one or more of the following clinical features: prostration (Blantyre Coma Score of 3 or 4), cerebral malaria (Blantyre Coma Score of <3), or respiratory distress (abnormally deep breathing).^7^ Cases were further classified on the basis of the results of blood tests taken on admission to hospital, including base deficit and haemoglobin concentration. Specifically, severe malaria anaemia was defined as a haemoglobin concentration of less than 50 g/L in association with respiratory distress. Finally, cases were also classified on the basis of their hospital inpatient survival status. Controls were children aged 3–12 months who were born within the same study area between August, 2006, and September, 2010, and who were recruited to an ongoing cohort study investigating genetic susceptibility to a range of childhood diseases.^8^

The design of the cohort study has been described in detail previously.^9^ In brief, a cohort of children and adults of all ages was recruited from a representative sample of households within the Ngerenya and Chonyi areas of Kilifi County during August, 1998, and enriched thereafter through the recruitment of children born subsequently into study homesteads. Participants were visited once each week by trained fieldworkers who monitored for febrile episodes through inquiry about their health during the preceding interval and by measuring axillary temperature. If participants reported an illness, they were encouraged to attend a dedicated outpatient clinic staffed by study clinicians with access to a wide range of quality-assured diagnostic tests. The following case definitions were applied: malaria was defined as an episode of fever (axillary temperature >37·5°C) in association with a slide positive for blood-stage asexual *P falciparum* parasites of any density; non-malaria fever was defined as an episode of fever in a child whose slide was negative for *P falciparum*; upper respiratory tract infection was diagnosed in children whose principal symptoms were characterised by rhinitis or pharyngitis and who had no other features of malaria; lower respiratory tract infection was diagnosed in children who fulfilled the WHO clinical criteria for pneumonia if tests and the subsequent clinical course of disease supported this diagnosis; gastroenteritis was defined as diarrhoea (≥three watery stools per day) with or without vomiting (≥three episodes per day); skin infection was diagnosed in children who presented with dermatological conditions, including scabies, boils, and impetigo; helminth infection was diagnosed in children who had a history of passing worms of any species; and severe anaemia was defined as a haemoglobin concentration of less than 50 g/L. The present analysis focuses on 752 children aged younger than 10 years between Aug 1, 1998, and July 31, 2001. Four cross-sectional surveys were done during this period through which the prevalence of *P falciparum* infection was recorded. Asymptomatic malaria parasitaemia was defined as the presence of a positive *P falciparum* blood film during these cross-sectional surveys in the absence of fever or symptoms of clinical illness.

Written informed consent was provided by the parents of all study participants and ethical approval for both studies was granted by the Kenya Medical Research Institute National Ethical Review Committee in Nairobi.

### Procedures

Haematological, biochemical, and malaria parasite data were derived as previously described.^10^ We tested for the G6PD c.202T allele and the G6PD c.376A allele, which is associated with a minor reduction in G6PD activity,^5^ on the Sequenom MassARRAY iPLEX platform (Agena Biosciences, Hamburg, Germany),^7^ using DNA extracted from fresh or frozen samples of whole blood by proprietary methods (ABI PRISM, Applied Biosystems, CA, USA; or Qiagen DNA Blood Mini Kit, Qiagen, West Sussex, UK). Because previous studies have suggested the possibility of epistasis with regard to malaria risk, between G6PD deficiency and both the sickle cell trait (haemoglobin genotype AS)^11^ and α thalassaemia,^12^, ^13^, ^14^ samples were also typed at these loci.^7^, ^15^

### Statistical analysis

The primary outcome measure for the case-control study was the odds ratio for hospital admission with severe malaria whereas for the cohort study it was the incidence rate ratio for uncomplicated malaria and non-malaria illnesses, by G6PD deficiency category.

For these analyses, we categorised study participants into the three most physiologically meaningful G6PD categories based on G6PD c.202T genotypes: (1) biochemically normal individuals—ie, boys or girls who were either hemizygotes or homozygotes for the wild-type allele at position 202 of the G6PD gene; (2) G6PD c.202T heterozygous girls (who have intermediate G6PD activity^5^); and (3) G6PD c.202T hemizygous boys and homozygous girls with G6PD deficiency. G6PD c.202T hemizygous boys and homozygous girls with G6PD deficiency have physiologically identical G6PD activity (roughly 12% of normal activity).^5^, ^16^

We stratified these groups by sex and by genotype at the G6PD 376 locus in secondary analyses. We compared continuous data using Student's *t* test, after normalisation by log-transformation where appropriate, whereas we compared proportions with the χ^2^ test. We calculated odds ratios for severe malaria and its sub-phenotypes in the case-control study by comparison of gene frequencies among severe malaria cases (both overall and by specific sub-phenotypes) with those among controls using logistic regression. We calculated genotype-specific incidence rate ratios (IRRs) for malaria and other diseases in the cohort study using Poisson regression analysis. We did all analyses both with and without adjustment for potential confounders. We tested for interactions between explanatory variables in both studies using the likelihood ratio test. We did all analyses using Stata (version 11.2).

### Role of the funding source

None of the funders had a role in the design of the study, the collection, analysis, or interpretation of the data, the writing of the report, or the decision to submit for publication. SU and CMN had full access to the raw data and TNW had full access to all of the data and had the final responsibility to submit for publication.

## Results

The case-control study included 2220 children with severe *P falciparum* malaria and 3940 control participants. Table 1 shows the demographic and laboratory characteristics of cases and controls, stratified by G6PD deficiency category, while table 2 shows the distribution of severe malaria syndromes by sex and G6PD deficiency. The G6PD c.202T allele was in Hardy-Weinberg equilibrium in the control population (χ^2^ p=0·32) and both cases and controls were well balanced with regard to sex (table 1). The proportion of G6PD c.202T heterozygous girls was lower among cases than controls (table 1) and was associated with a significantly reduced risk of severe malaria overall (adjusted odds ratio [OR] 0·82, 95% CI 0·70–0·97; p=0·020) and malaria inpatient death (table 3). A subgroup analysis of severe malaria phenotypes showed that the most likely protective effects were for cerebral malaria admission and cerebral malaria death (table 3). By contrast, the adjusted odds ratio for severe malaria in children with G6PD deficiency (homozygous girls and hemizygous boys for the G6PD c.202T allele) was 1·18 (95% CI 0·99–1·40; p=0·056) and the increased risk was significant for severe malaria anaemia (table 3). This increased risk in severe anaemia was associated with lower haemoglobin concentrations in homozygous girls and hemizygous boys with G6PD deficiency and severe malaria at the time of hospital admission (table 1). We found no significant evidence for an independent effect of the G6PD c.376A allele on the risk of severe malaria or syndrome-specific inpatient mortality (appendix p 2–4). Moreover, we found no evidence of an interaction between G6PD deficiency category and either sickle cell trait (57 cases and 596 controls had sickle cell trait; likelihood ratio test 0·19) or α-thalassaemia genotype (1026 cases and 1953 controls were heterozygotes for α thalassaemia and 264 cases and 637 controls were homozygotes; 0·61). Further analyses of the data for male and female participants separately made no material difference to these conclusions (appendix p 5).

Median follow-up for the mild disease cohort study was 2·24 years (IQR 2·22–2·85). We found no evidence of a significant association between G6PD deficiency category and the incidence of uncomplicated *P falciparum* malaria or other common diseases of childhood with the exception of a marginally significant increase in the incidence of helminth infections in heterozygous girls (table 4). We detected no significant differences in densities of *P falciparum* parasites during asymptomatic, uncomplicated, and severe clinical infections by G6PD category (table 5).

## Discussion

Through a case-control study done on the coast of Kenya, we found that girls who were heterozygotes for the G6PD c.202T allelic form of G6PD deficiency were significantly protected from severe and complicated *P falciparum* malaria. Conversely, children with G6PD deficiency had a significantly increased risk of severe malaria anaemia. We found no effect of G6PD category on parasite densities or on the incidence of uncomplicated *P falciparum* malaria or other common diseases of childhood either in the same study or in a cohort study of children living in the same area. The only exception we found was a marginally significant increase in the incidence of helminth infections among heterozygous girls, an observation which may well represent a chance finding given the number of comparisons we made.

The global distribution of G6PD deficiency is thought to be a result of natural selection through providing a survival advantage against malaria.^17^ Nevertheless, clinicoepidemiological studies have resulted in a confusing picture of which, if any, of the alleles responsible for G6PD deficiency protect against malaria, against which malaria species, and against which of the many clinical manifestations of severe malaria. Whether any such advantage is exclusive to heterozygous female individuals, or whether it is shared by both sexes equally has been especially controversial.^4^, ^18^ Many factors might account for these apparently conflicting conclusions, including small sample sizes, variations in study design and malaria outcomes, inconsistent definitions for G6PD deficiency (including those based on both biochemical and genetic methods), allelic heterogeneity at the G6PD locus, and epistatic interactions between G6PD deficiency and polymophisms at other loci. In a particularly influential study done in The Gambia and Kenya,^19^ the authors concluded that both G6PD deficient hemizygous boys and heterozygous girls were protected from severe malaria. However, the genetic basis of G6PD deficiency in The Gambia is more complex than previously assumed, with the result that this conclusion has since been questioned.^4^, ^18^ The result of these conflicting findings is that efforts to develop new malaria treatments on the basis of pathophysiological studies of G6PD deficiency remain somewhat speculative.

We investigated the effect of G6PD deficiency both on a range of phenotypes of clinical malaria and on the incidence of other common diseases of childhood within a single well-characterised population. Based on data from our case-control study, the risks of severe malaria and within-hospital death among G6PD c.202T heterozygous girls were significantly lower than in G6PD normal children. Conversely, the risks of severe malarial anaemia in G6PD c.202T hemizygous boys and homozygous girls with G6PD deficiency were significantly higher. Data from our cohort study showed no effect of G6PD category on the prevalence or incidence of either uncomplicated malaria or of any other common childhood disease, with the sole exception of a marginally significant increase in the incidence of helminth infections among heterozygous girls. Taken together, our data support the previous conclusion that G6PD deficiency is a balanced polymorphism conferring a selection advantage in heterozygous girls at the expense of a fitness disadvantage in those with G6PD deficiency.^4^, ^18^

Although our conclusions support the results of some previous clinical studies,^20^, ^21^, ^22^, ^23^, ^24^ they do not concur with others that have found either no association between G6PD deficiency and various phenotypes of *P falciparum* infection,^25^, ^26^ or evidence for protection in various combinations of males and females^19^, ^27^, ^28^ in several other settings. Moreover, *P falciparum* is the only significant cause of clinical malaria in Kenya; therefore, we were unable to investigate the hypothesis that G6PD deficiency might protect against *Plasmodium vivax* malaria.^29^ Nevertheless, we believe that our study, which to the best of our knowledge is the largest and most comprehensive yet reported from a single site, has several advantages over many previous studies.

First, we characterised participants on the basis of G6PD genotype as opposed to G6PD phenotype. The high degree of overlap in enzyme activity between the various haplotypic groups^5^, ^30^ and the fact that almost all the allelic sites in the common haplotypes have no known or measurable effect on G6PD activity makes it impossible to predict G6PD genotypes from data for G6PD enzyme activity alone. This limitation is particularly relevant for polymorphisms that confer milder phenotypes, such as the common African G6PD c.202T and G6PD c.376A polymorphisms in which, for example, there is no overlap in G6PD activity between male hemizygotes with the G6PD c.202T allele and those with the G6PD c.376A allele, the latter being essentially G6PD normal.^31^ For this reason, with one exception,^20^ phenotypic studies have classified participants as normal or deficient on the basis of arbitrary break points that may or may not be biologically meaningful. Although only four alleles have been found to be associated with G6PD deficiency in African populations (G6PD c.202T, G6PD c.542A, G6PD c.680T, and G6PD c.968C^32^, ^33^), studies have not been exhaustive and other alleles might remain to be discovered. As such, we believe that a genetic approach to the classification of G6PD deficiency is likely to yield more interpretable results.

A second strength of our study is that, by contrast with most previous studies in which participants have been selected on the basis of G6PD genotype, we minimised the possibility of misclassification of G6PD deficiency through earlier studies^32^ in which we established that the G6PD c.202T allele is the only substantial determinant of G6PD deficiency in our study population.^5^ Interpreting data from studies done in areas in which G6PD activity is affected by multiple polymorphisms is much more challenging. Under such circumstances, unrecognised allelic heterogeneity can lead to confounding through misclassification of participants as G6PD normal, which might have led to misleading conclusions in previous studies done in west Africa, where there is greater diversity at the G6PD locus.^4^, ^18^ Even when recognised, allelic heterogeneity can make the interpretation of epidemiological studies more difficult because different mutations can have widely differing phenotypic effects^1^ that can make classifications based on combined groupings somewhat hypothetical. The situation is further complicated by the fact that G6PD deficiency seems to have two opposing actions on severe malaria, decreasing the risk of cerebral malaria but increasing the risk of severe malarial anaemia, as shown in a large multicentre study that included some of the data reported here.^7^ As a consequence, the overall effect of G6PD deficiency might depend on the relative frequencies of cerebral malaria and severe malaria anaemia, which is related to the intensity of malaria transmission and will therefore vary over time and between different epidemiological settings. Finally, the alternative approach of analysing studies by individual genotypes, stratified by sex, leads to difficulties with analytical power, as exemplified by a study done in The Gambia, despite using a large sample.^32^

Unlike most previous studies, we excluded the possibility of confounding by two other malaria-protective polymorphisms—sickle cell trait and α thalassaemia—both of which are common in our study population. A negative epistatic interaction between sickle cell trait and G6PD deficiency has been reported previously in Mali,^11^ whereas the possibility of epistasis with the thalassaemias has been suggested by population genetic studies in Sardinia^12^, ^13^ and Saudi Arabia.^14^ Nevertheless, data from our study show that in Kilifi, the effect of G6PD deficiency on malaria is independent from both these polymorphisms, and our findings are robust to adjustments for these independent effects. Finally, we studied the effect of G6PD deficiency on both malaria-specific and non-malaria-specific outcomes within a single well-characterised population, and found no evidence for a significant effect of G6PD deficiency category on susceptibility to uncomplicated clinical malaria or to other common diseases of childhood.

Despite having arisen thousands of years ago, there remain no examples of populations in which any of the many G6PD deficiency mutations have been selected to the point of fixation,^4^ suggesting that either their selective advantage is very small or that their rising frequency has been checked by balancing selection. Our findings support the latter hypothesis: that frequencies of the G6PD c.202T allele in Kilifi are a result that frequencies of the G6PD c.202T allele in Kilifi are a result of historic selection for heterozygous girls through a survival advantage against severe malaria balanced by the increased loss from severe malaria anaemia of both G6PD c.202T hemizygous boys and homozygous girls with G6PD deficiency. The G6PD c.202T allele is associated with such a mild phenotype that even hemizygous boys and homozygous girls retain 12% of normal G6PD activity^5^, ^34^ and, as a result, are rarely affected by the more severe manifestations of G6PD deficiency, with the exception of challenge by powerful oxidants such as dapsone.^35^ We suggest, therefore, that historically, malaria alone may be sufficient to explain balancing selection against G6PD c.202T hemizygous boys and homozygous girls.

Our study has several limitations of which perhaps the most important relates to the fact that, to a large extent, our study design was opportunistic and capitalised on the existence of data and samples collected for other purposes. As a result, participants were recruited over a long period during which the transmission of malaria within the study area declined substantially.^36^ Moreover, our control participants were substantially younger than case participants. However, we believe that such design limitations would be more likely to mitigate against our results than favour them because any selection for G6PD deficiency by malaria would result in a rising prevalence by age and over time, which would have the effect of enhancing rather than reducing the differences that we have recorded.

Second, the lack of statistical evidence for an effect of G6PD deficiency on the incidence of uncomplicated malaria or other childhood diseases might simply be a result of a lack of statistical power, either because the study or the effect sizes were too small. Furthermore, being purely epidemiological, our study can provide no definitive insights into the mechanisms by which G6PD deficiency confers malaria protection. Nevertheless, the fact that we found no meaningful difference in parasite densities between G6PD deficiency categories across the full range of *P falciparum* infections does not, on the face of it, support most hypotheses advanced to date. Most have invoked the reduced ability of erythrocytes of people with G6PD deficiency to cope with the oxidant stresses that accompany *P falciparum* infection, and suggest that this effect might either reduce the ability of parasites to grow and develop or precipitate their early removal once infected.^3^, ^37^ Under either of these scenarios, we would anticipate that children with G6PD deficiency would have lower parasite densities during clinical infections in vivo. Although reduced densities were noted in several early studies, which are often cited in support of existing mechanistic hypotheses,^20^, ^21^, ^38^ more recent studies have been inconsistent, with some showing lower densities in various subgroups,^39^, ^40^ some finding none,^28^, ^41^ and most suffering from low statistical power. Therefore, it can be said only that the mechanism of protection remains unproven and is worthy of additional research.

Why G6PD deficiency should be associated with an increased risk of severe malaria anaemia is also open to speculation; however, malaria parasites exert oxidant stress on red cells^42^ and excessive oxidant stress causes acute haemolysis in people with G6PD deficiency,^1^ meaning that the excess risk of anaemia during the course of malaria infections in children with G6PD deficiency makes biological sense. Similarly, the selective advantage for heterozygous girls might be explained by the fact that, as a result of random inactivation of the X chromosome, they benefit from two roughly equal populations of red blood cells—one fully deficient and the other fully non-deficient.^20^ This raises the possibility that such children enjoy the best of both worlds: roughly half of their red blood cells being protected by G6PD deficiency through a mechanism that remains incompletely understood, while the remaining half buffer against an overwhelming drop in haemoglobin that can complicate malaria more often in fully deficient individuals. Although it has been suggested that more subtle changes in G6PD structure or function, including that associated with the G6PD c.376A allele, might also confer protection against malaria,^24^ this possibility is not supported by data from this study, which show that the effects of G6PD on malaria risk were exclusively related to the G6PD c.202T mutation.

In conclusion, more than half a century since the protective effect of G6PD deficiency against malaria was first proposed, the clinical epidemiology of malaria in children with G6PD deficiency has remained confusing. Our study provides strong support for the conclusion that the G6PD c.202T allele has reached its present frequencies in much of sub-Saharan Africa through positive selection for heterozygous girls through a mechanism mediated by malaria. We hope that this growing consensus will lead to a renewed drive to determine the mechanisms involved and to turn this knowledge to new approaches to malaria treatment.

## Figures and Tables

**Table 1:** Characteristics of patients in the case-control study

		**G6PD normal boys and girls**	**G6PD c.202T heterozygous girls**	**G6PD c.202T homozygous girls and hemizygous boys**	**p value**
Controls		2863 (73%)	639 (16%)	438 (11%)	
	Median age (IQR; months)	6·3 (6·2–6·4)	6·4 (6·2–6·6)	6·4 (6·2–6·6)	0·68
Cases		1643 (74%)	306 (14%)	271 (12%)	
	Median age (IQR; months)	24·7 (23·7–25·6)	23·0 (21·0–25·2)	25·3 (23·1–27·7)	0·31
	Mean haemoglobin (95% CI; g/L)	62 (60–63)	62 (58–65)	57 (54–60)	0·0071
	Mean MCV (95% CI; fL)	73·2 (72·7–73·6)	75·1 (74·0–76·2)	74·3 (73·1–75·5)	0·0008
	Mean platelets (95% CI; × 10^6^/L)	112 (107–117)	123 (111–136)	134 (120–150)	0·014
	Mean base deficit (mM)	8·5 (8·1–8·9)	8·9 (8·0–9·9)	7·9 (7·0–8·9)	0·13

p values estimated by Kruskal-Wallis rank test. MCV=mean corpuscular volume.

**Table 2:** Distribution of clinical syndromes of severe malaria among cases within the case-control study

	**All**	**G6PD normal boys and girls**	**G6PD c.202T heterozygous girls**	**G6PD c.202T homozygous girls and hemizygous boys**
Severe malaria admissions*				
Cerebral malaria	1220/2064 (59%)	921/1534 (60%)	168/283 (59%)	131/247 (53%)
Respiratory distress	680/2154 (32%)	510/1601 (32%)	98/294 (33%)	72/259 (28%)
Severe malaria anaemia	683/2220 (31%)	479/1643 (29%)	90/306 (29%)	111/271 (41%)
Other†	374/2220 (17%)	281/1525 (18%)	47/282 (17%)	46/244 (19%)
**Inpatient death**‡				
Overall	256/2220 (12%)	207/1634 (13%)	26/306 (8%)	23/270 (9%)
Cerebral malaria	192/1220 (16%)	155/921 (17%)	19/168 (11 %)	18/131 (14%)
Respiratory distress	117/680 (17%)	97/510 (19%)	11/98 (11%)	9/72 (13%)
Severe malaria anaemia	81/683 (12%)	64/479 (13%)	9/90 (10%)	8/111 (7%)
Other†	21/374 (6%)	16/281 (6%)	2/47 (4%)	3/46 (7%)

Children can appear in more than one category.

* Data are missing for some syndromes.

† Severe malaria cases without any of the other primary characteristics (or when data for these characteristics are missing).

‡ Denotes the number of deaths among case patients displaying specific clinical features.

**Table 3:** Risk of severe malaria and inpatient death in the case-control study

	**G6PD c.202T heterozygous girls**		**G6PD c.202T homozygous girls and hemizygous boys**	
	Odds ratio (95% CI)	p value	Odds ratio (95% CI)	p value
**Admission to hospital**				
All severe malaria	0·82 (0·70–0·97)	0·020	1·18 (0·99–1·40)	0·056
Cerebral malaria	0·81 (0·67–0·98)	0·037	0·98 (0·78–1·22)	0·88
Respiratory distress	0·84 (0·66–1·07)	0·17	1·00 (0·76–1·32)	0·96
Severe anaemia	0·86 (0·66–1·10)	0·24	1·71 (1·34–2·18)	<0·0001
Other severe malaria	0·76 (0·56–1·04)	0·088	1·18 (0·86–1·62)	0·29
**Inpatient death**				
Overall	0·54 (0·35–0·84)	0·0070	0·76 (0·48–1·20)	0·24
Cerebral malaria	0·51 (0·30–0·86)	0·011	0·99 (0·96–1·01)	0·37
Respiratory distress	0·48 (0·25–0·94)	0·032	0·66 (0·33–1·34)	0·26
Severe anaemia	0·58 (0·27–1·23)	0·161	0·78 (0·35–1·74)	0·56
Other severe malaria	0·48 (0·25–0·94)	0·032	0·66 (0·33–1·34)	0·26

Odds ratios are from comparisons of allele frequencies in cases versus controls by logistic regression with adjustment for sickle cell trait and α-thalassaemia genotype and ethnic group. Children could contribute data to more than one category.

**Table 4:** Incidence of malaria and other common childhood diseases in the mild-disease cohort study

	**Episodes**	Incidence*	**IRR (95% CI)**†	**p value**
**All diagnoses**				
G6PD normal boys and girls	5830	5·22	1	
G6PD c.202T heterozygous girls	1314	5·51	0·98 (0·86–1·11)	0·82
G6PDd boys and girls	1149	4·96	0·93 (0·82–1·04)	0·25
**Malaria**				
G6PD normal boys and girls	2159	1·93	1	
G6PD c.202T heterozygous girls	508	2·13	1·09 (0·86–1·38)	0·45
G6PDd boys and girls	440	1·90	0·90 (0·74–1·10)	0·34
**Non-malaria fever**				
G6PD normal boys and girls	615	0·55	1	
G6PD c.202T heterozygous girls	130	0·54	1·01 (0·80–1·28)	0·91
G6PDd boys and girls	117	0·50	1·00 (0·77–1·29)	0·97
**URTI**				
G6PD normal boys and girls	1610	1·44	1	
G6PD c.202T heterozygous girls	379	1·59	1·03 (0·87–1·21)	0·68
G6PDd boys and girls	290	1·25	0·93 (0·78–1·10)	0·41
**LRTI**				
G6PD normal boys and girls	636	0·56	1	
G6PD c.202T heterozygous girls	136	0·57	0·77 (0·56–1·05)	0·11
G6PDd boys and girls	113	0·48	0·99 (0·76–1·30)	0·98
**Gastroenteritis**				
G6PD normal boys and girls	574	0·51	1	
G6PD c.202T heterozygous girls	113	0·47	0·94 (0·72–1·24)	0·70
G6PDd boys and girls	113	0·48	1·16 (0·93–1·45)	0·18
**Skin infection**				
G6PD normal boys and girls	675	0·60	1	
G6PD c.202T heterozygous girls	166	0·69	1·16 (0·85–1·57)	0·33
G6PDd boys and girls	152	0·65	1·18 (0·89–1·57)	0·22
**Helminth infection**				
G6PD normal boys and girls	479	0·42	1	
G6PD c.202T heterozygous girls	131	0·54	1·32 (1·00–1·74)	0·048
G6PDd boys and girls	100	0·43	1·17 (0·88–1·54)	0·26
**Severe anaemia**				
G6PD normal boys and girls	18	0·01	1	
G6PD c.202T heterozygous girls	3	0·01	0·29 (0·03–2·33)	0·25
G6PDd boys and girls	3	0·01	0·35 (0·04–2·54)	0·30

Data are for 531 G6PD normal girls and boys (1100 person-years of follow-up), 115 G6PD c.202T heterozygous girls (238 person-years of follow-up), and 106 G6PD c.202T homozygous girls or hemizygous boys (231 person-years of follow-up). URTI=upper respiratory tract infection. LRTI=lower respiratory tract infection.

* Episodes per year of follow-up.

† Adjusted for age, location, haemoglobin S genotype, and α-thalassaemia genotype.

**Table 5:** *Plasmodium falciparum* densities in the case-control and mild-disease cohort studies

	**Asymptomatic**			**Uncomplicated**			**Complicated**		
	N	Mean (95% CI)	p value	N	Mean (95% CI)	p value	N	Mean (95% CI)	p value
G6PD normal boys and girls	446	1430 (1207–1695)	..	1152	23 024 (20 283–26 136)	..	1643	41 464 (36 781–46 742)	..
G6PD c.202T heterozygous girls	88	1195 (830–1722)	..	246	20 051 (15 117–26 595)	..	306	41 157 (31 184–54 319)	..
G6PD c.202T homozygous girls and hemizygous boys	113	1703 (1185–2448)	0·33	251	20 031 (15 144–26 495)	0·56	271	35 063 (26 432–46 512)	0·39

We measured asymptomatic parasite densities through cross-sectional surveys of members of the mild-disease cohort study (data collected from 321 G6PD normal girls and boys, 81 G6PD c.202T heterozygous girls, and 81 G6PD c.202T homozygous girls or hemizygous boys). We assessed densities during uncomplicated malaria infections recorded within the mild-disease cohort study (data contributed by 409 G6PD normal girls and boys [856 person-years of follow-up], 87 G6PD c.202T heterozygous girls [187 person-years of follow-up], and 85 G6PD c.202T homozygous girls or hemizygous boys [180 person-years of follow-up]). We assessed densities during complicated malaria episodes in severe malaria cases within the case–control study. We used the Kruskal-Wallis rank test to calculate p values to compare the three groups within each category.
