# Supplementary material for: Glucose-6-phosphate dehydrogenase deficiency and the risk of malaria and other diseases in children in Kenya: a case-control and a cohort study
Source: Lancet Haematol. 2015 Sep 22;2(10):e437–44. doi: 10.1016/S2352-3026(15)00152-0 (PMC4703047; doi:10.1016/S2352-3026(15)00152-0)
Supplement: Supplementary appendix [file mmc1.pdf]

THE LANCET Haematology

## Supplementary appendix

This appendix formed part of the original submission and has been peer reviewed. We post it as supplied by the authors.

Supplement to: Uyoga S, Ndila CM, et al, for the MalariaGEN Consortium. Glucose-6-phosphate dehydrogenase deficiency and the risk of malaria and other diseases in children in Kenya: a case-control and a cohort study. *Lancet Haematol* 2015; published online Sept 23. http://dx.doi.org/10.1016/S2352-3026(15)00152-0.

## Web Extra Material.

**Figure. Case-control study profile**

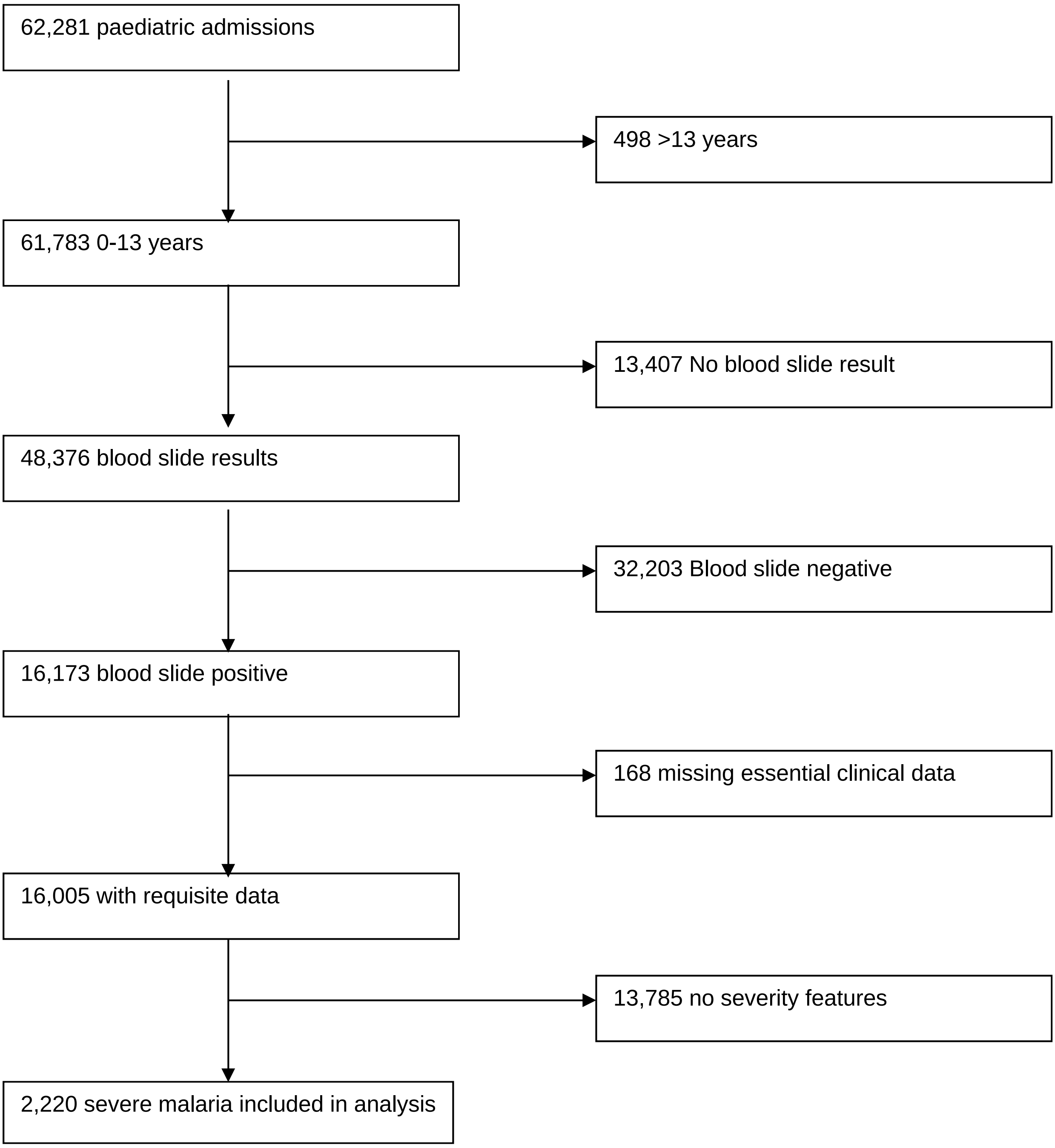

**Table 1. Odds ratios for severe malaria, stratified by G6PD haplotype at the 376 and 202 loci.**

| G6PD haplotype | Cases | Controls | OR (95% CI) | *P* | aOR* (95% CI) | *P* |
|---|---|---|---|---|---|---|
| WT/WT | 1,046 | 1,857 | 1 | | 1 | |
| WT/376 | 273 | 487 | 0.99 (0.84-1.17) | 0.80 | 1.00 (0.84-1.19) | 0.96 |
| 376/376 | 314 | 506 | 1.10 (0.93-1.29) | 0.42 | 1.12 (0.94-1.32) | 0.18 |
| WT/202 | 218 | 471 | 0.82 (0.68-0.98) | 0.03 | 0.80 (0.67-0.97) | 0.031 |
| 376/202 | 85 | 167 | 0.90 (0.68-1.18) | 0.47 | 0.93 (0.70-1.25) | 0.67 |
| 202/202 | 271 | 432 | 1.11 (0.93-1.31) | 0.21 | 1.22 (1.02-1.46) | 0.030 |

WT/WT: male hemizygotes and female homozygotes wild type at both the 376 and 202 loci.
WT/376: female heterozygotes for the 376G allele and WT at the 202 allele.
376/376: male hemizygotes and female homozygotes for the 376G allele and WT at the 202 locus.
WT/202: female heterozygotes for the 202 allele.
376/202: compound heterozygous females for both 376G and 202A.
202/202: male hemizygotes and female homozygotes for 202A.
* ORs adjusted for HbS and $\alpha$-thalassaemia genotype and ethnic group.
LR test for interaction between 202 and 376 p=0.84

.

**Table 2. OR for in-patient death by G6PD haplotype**

| G6PD haplotype[$] | Severe malaria admissions | Deaths (%) | aOR (95% CI)* | *P* |
|---|---|---|---|---|
| WT/WT | 1,046 | 137 (13.10) | 1 | |
| WT/376 | 273 | 33 (12.13) | 0.88 (0.58-1.32) | 0.54 |
| 376/376 | 314 | 37 (11.78) | 1.011 (0.68-1.48) | 0.95 |
| WT/202 | 218 | 17 (7.80) | 0.48 (0.28-0.81) | 0.0070 |
| 376/202 | 85 | 9 (10.59) | 0.70 (0.33-1.46) | 0.35 |
| 202/202 | 271 | 23 (8.49) | 0.75 (0.47-1.20) | 0.24 |

* ORs adjusted for HbS and α-thalassaemia genotype and ethnic group.
[$] G6PD haplotypes defined as in Supplementary Table 1.

**Table 3. OR for in-patient death by G6PD haplotype, stratified by severe malaria phenotype[a].**

| G6PD haplotype[$] | Deaths | aOR* (95% CI) | *P* |
|---|---|---|---|
| *Cerebral malaria* [a] | | | |
| WT/WT | 108 | 1 | |
| WT/376 | 22 | 0.73 (0.44-1.20) | 0.22 |
| 376/376 | 25 | 0.84 (0.53-1.34) | 0.48 |
| WT/202 | 11 | 0.37 (0.19-0.72) | 0.0041 |
| 376/202 | 8 | 0.77 (0.35-1.70) | 0.53 |
| 202/202 | 18 | 0.43 (1.02-1.25) | 0.27 |
| *Severe malaria anaemia* [a] | | | |
| WT/WT | 39 | 1 | |
| WT/376 | 13 | 1.14 (0.57-2.26) | 0.70 |
| 376/376 | 12 | 1.16 (0.59-2.25) | 0.66 |
| WT/202 | 7 | 0.62 (0.26-1.49) | 0.29 |
| 376/202 | 2 | 0.59 (0.14-2.52) | 0.48 |
| 202/202 | 2 | 0.84 (0.37-1.90) | 0.68 |
| *Respiratory distress* [a] | | | |
| WT/WT | 67 | 1 | |
| WT/376 | 15 | 0.84 (0.46-1.52) | 0.58 |
| 376/376 | 15 | 0.79 (0.43-1.43) | 0.45 |
| WT/202 | 7 | 0.36 (0.15-0.85) | 0.02 |
| 376/202 | 4 | 0.70 (0.25-1.97) | 0.51 |
| 202/202 | 9 | 0.63 (0.31-1.29) | 0.30 |

[$] G6PD haplotypes defined as in Supplementary Table 1.
[a] Some patients manifest features of more than one phenotype.
* ORs adjusted for HbS and $\alpha$-thalassaemia genotype and ethnic group.

**Table 4. Odds ratios for all severe malaria and severe malaria syndromes by G6PD 202 genotype and gender.**

| | **Heterozygous females** | | **Homozygous females** | | **Hemizygous males** | |
|---|---|---|---|---|---|---|
| | **(95% CI)** | ***P**** | **(95% CI)** | ***P**** | **(95% CI)** | ***P**** |
| All severe malaria adjusted | 0.82 (0.68-0.96) | 0.020 | 1.20 (0.78-1.85) | 0.40 | 1.18 (0.98-1.44) | 0.077 |
| Severe malaria syndromes [a] | | | | | | |
| Coma | 0.79 (0.64-0.98) | 0.039 | 1.21 (0.72-2.04) | 0.46 | 0.95 (0.74-1.22) | 0.71 |
| Respiratory distress | 0.87 (0.66-1.13) | 0.32 | 1.22 (0.64-2.34) | 0.54 | 0.94 (0.69-1.29) | 0.74 |
| Severe malaria anaemia | 0.81 (0.61-1.07) | 0.15 | 1.14 (0.58-2.26) | 0.69 | 1.88 (1.43-2.47) | <0.001 |
| Other SM | 0.74 (0.52-1.03) | 0.080 | 1.20 (0.55-2.61) | 0.63 | 1.19 (0.83-1.70) | 0.33 |
| Fatal outcome | 0.60 (0.37-0.96) | 0.033 | 0.25 (0.03-1.87) | 0.18 | 0.79 (0.49-1.29) | 0.36 |

[a] Some patients manifest features of more than one phenotype.
* ORs adjusted for HbS and $\alpha$-thalassaemia genotype and ethnic group.
The analysis was based on the following denominators:
Controls: females 1951 (1250 normals, 639 heterozygotes and 62 homozygotes); males 1989 (1613 normals, 376 hemizygotes).
Cases: females 1074 (729 normals, 306 heterozygotes, 39 homozygotes); males 1146 (914 normals, 232 hemizygotes).
